# Worldwide Reduction in MERS Cases and Deaths since 2016

**DOI:** 10.3201/eid2509.190143

**Published:** 2019-09

**Authors:** Christl A. Donnelly, Mamun R. Malik, Amgad Elkholy, Simon Cauchemez, Maria D. Van Kerkhove

**Affiliations:** University of Oxford, Oxford, UK (C.A. Donnelly);; Imperial College London, London, UK (C.A. Donnelly);; World Health Organization Regional Office for the Eastern Mediterranean, Cairo, Egypt (M.R. Malik, A. Elkholy);; Institut Pasteur, Paris, France (S. Cauchemez);; World Health Organization, Geneva, Switzerland (M.D. Van Kerkhove)

## Abstract

Since 2012, Middle East respiratory syndrome (MERS) coronavirus has infected 2,442 persons worldwide. Case-based data analysis suggests that since 2016, as many as 1,465 cases and 293–520 deaths might have been averted. Efforts to reduce the global MERS threat are working, but countries must maintain vigilance to prevent further infections.

From 2012 through May 31, 2019, Middle East respiratory syndrome coronavirus (MERS-CoV) has infected 2,442 persons and killed 842 worldwide (*1*). MERS-CoV is currently circulating in dromedary camels in Africa, the Middle East, and southern Asia; however, most cases of human infection have been reported in the Arabian Peninsula (*2*). Large hospital outbreaks in 2014 and 2015 (*3**,**4*) (Appendix Figure 1) motivated affected countries to substantially invest in prevention and control activities. 

To estimate the potential number of MERS cases and deaths that might have been averted since 2016 had the risk levels of 2014–2015 continued, we analyzed case-based data on laboratory-confirmed human cases of MERS-CoV infections reported to the World Health Organization (*5*). We categorized cases as either secondary (human-to-human transmission) or community-acquired (presumed camel-to-human transmission). In addition, we used case-based data on date of onset (for symptomatic infections) or report (for asymptomatic infections), outcome (died/recovered), and dates and sizes of reported clusters of human-to-human–transmission cases (*3**,**4**,**6**–**8*).

We compared incidence of camel-to-human–transmission cases (i.e., community-acquired cases, assuming all of those not positively attributed to human-to-human transmission were in this category) during 2016, 2017, and 2018 (through September only) with incidence during 2014–2015, assuming that case numbers were Poisson distributed (yielding a 2-sided p value). Furthermore, we obtained the expected total number of cases in 2016, 2017, and through September 2018, conditional on the incidence of community-acquired cases, by simulating 10,000 times from the distribution of human-to-human–transmission cluster sizes observed during 2014–2015. Thus, the observed incidence rates in these years could be compared with simulations to test the null hypothesis that human-to-human transmission levels remained constant since 2014–2015 (yielding a 2-sided p value). The intervals reported are the 2.5th and 97.5th percentiles of the simulations (95% CIs). We examined a range of mortality rates from healthcare-associated outbreaks in South Korea and Saudi Arabia (*3**,**5*) and the case-fatality ratio (CFR) from all reported cases globally (35.5%, 800 fatalities/2,254 cases) (*9*)*.* When numbers of cases averted were not statistically significant, we truncated the lower bound of the 95% CI to 0 cases averted.

Of the 2,254 laboratory-confirmed cases reported to the World Health Organization from 2012 through October 1, 2018 (Appendix Figure 1), 1,087 were classified as human-to-human transmssion cases and the remaining 1,167 as community-acquired cases. During this same period, clusters/outbreaks were reported each year (range 2–255 cases).

Although 739 cases were reported in 2014 and 768 cases in 2015, only 244 cases were reported in 2016, another 244 in 2017, and 113 through September 2018. We assessed potential components of this reduction (i.e., reduction of community-acquired cases, human-to-human transmission cases, or both). The incidence of community-acquired cases was 177 in 2016, 151 in 2017, and 86 through September 2018 (Appendix Table). These rates were each significantly (p<0.001) lower than expected compared with the incidence in 2014–2015 (334 for 2016, 334 for 2017, and 251 through September 2018). Conditional on the number of community-acquired cases, we observed no significant reduction in the risk for secondary cases from 2014–2015 to 2016, 2017, and through September 2018, although we did find nonsignificant trends. We estimated that 154 secondary cases (95% CI 0–495) were averted from the 177 community-acquired cases in 2016, 96 (95% CI 0–419) from the 151 community-acquired cases in 2017, and 80 (95% CI 0–338) from the 86 community-acquired cases through September 2018, totaling 330 (95% CI 0–819) from the 414 community-acquired cases during 2016–September 2018 (Table). Assuming a 20% CFR (*3**,**10*), these 330 (95% CI 0–819) cases averted correspond to 66 (95% CI 0–164) expected deaths averted; assuming a 35.5% CFR (*9*), they correspond to 117 (95% CI 0–291) expected deaths averted.

**Table:** Estimated Middle East respiratory syndrome cases and deaths averted because of reduced human-to-human transmission and camel-to-human transmission*

Year	Estimated cases and deaths averted because of reduced human-to-human transmission†					Estimated cases and deaths averted because of reduced camel-to-human and human-to-human transmission			
	Cases averted‡	2-sided p value	Deaths averted			Cases averted‡	2-sided p value	Deaths averted	
			Assuming 20% CFR‡	Assuming 35.5% CFR‡				Assuming 20% CFR‡	Assuming 35.5% CFR‡
2016	154 (0–495)	0.2714	31 (0–99)	55 (0–176)		507 (189–967)	<0.0001	101 (38–193)	180 (67–343)
2017	96 (0–419)	0.5810	19 (0–84)	34 (0–149)		507 (189–967)	<0.0001	101 (38–193)	180 (67–343)
2018§	80 (0–338)	0.4316	16 (0–68)	29 (0–120)		451 (191–855)	<0.0001	90 (38–171)	160 (68–304)
2016–2018§	330 (0–819)	0.0896	66 (0–164)	117 (0–291)		1,465 (895–2165)	<0.0001	293 (179–433)	520 (318–769)

*Values are estimated no. (95% range) except as indicated. CFR, case-fatality ratio. †Conditional on reported community-acquired cases. ‡The 95% intervals reported are the 2.5th and 97.5th percentiles of the simulations. When cases averted were not statistically significant, we truncated the lower bound of the 95% CI to 0 cases averted. §Through September 2018.

The total number of cases averted, when simultaneously taking into account reduced camel-to-human and human-to-human transmission, was estimated at 507 (95% CI 189–967) in 2016, 507 (95% CI 189–967) in 2017, and 451 (95% CI 191–855) through September 2018, totaling 1,465 (95% CI 895–2,165) cases averted and 293 (95% CI 179–433) expected deaths averted (under the assumption of a 20% CFR) from 2016 through September 2018. Assuming a 35.5% CFR, this estimate corresponds to 520 (95% CI 318–769) expected deaths averted.

We believe that affected countries are reducing the global threat of MERS by addressing knowledge gaps with regard to transmission, enhancing surveillance, and strengthening the ability to detect cases early and contain outbreaks through improved infection prevention and control measures in hospitals. Critical for preventing international spread and sustained transmission have been improved prevention and control measures in hospitals, restriction of camel movement in affected areas, stronger and more comprehensive investigations of cases and clusters, and improved communication.

Although global efforts seem to have prevented hundreds of infections and deaths, vigilance must be maintained by all countries. More needs to be done to limit spillover infections from dromedaries, which requires stronger surveillance of dromedary populations and persons in direct contact with infected herds and accelerated development of a vaccine for dromedaries (*2*). The international community and affected countries have a collective and shared responsibility to curtail a major health security threat such as MERS in the Middle East and beyond.

Appendix Supplementary information about study of worldwide reduction in MERS cases and deaths since 2016.
