# Supplementary material for: Worldwide Reduction in MERS Cases and Deaths since 2016
Source: Emerg Infect Dis. 2019 Sep;25(9):1758–60. doi: 10.3201/eid2509.190143 (PMC6711233; doi:10.3201/eid2509.190143)
Supplement: Appendix — Supplementary information about study of worldwide reduction in MERS cases and deaths since 2016. [file 19-0143-Techapp-s1.pdf]

Article DOI: https://doi.org/10.3201/eid2509.190143

Worldwide Reduction in MERS Cases and Deaths since 2016

Appendix

Background

While to date this high-threat zoonotic respiratory pathogen typically has not caused outbreaks in community settings, it has repeatedly demonstrated its ability to cause large-scale outbreaks in healthcare settings in several countries including the Kingdom of Saudi Arabia (KSA), the United Arab Emirates and the Republic of Korea (ROK) with substantial public health, security and economic impacts (Appendix Figure 1) (*1*).

The prevention and control measures invested in to reduce MERS-CoV incidence included better surveillance in dromedary camel (*1*,*2*) and human (*3*-*5*) populations, rapid and accurate detection of cases and human-to-human-transmission clusters, risk assessment, transparency and information sharing with affected countries, to WHO and externally, evidence-based policy development and revisions (*3*-*6*), and improvements in basic infection prevention and control measures in healthcare settings.

Data on human MERS-CoV cases

Within WHO databases, human cases were classified as secondary cases due to human-to-human transmission if they reported recent direct contact with a known MERS patient and/or were identified as a household, occupational or healthcare worker contact of a known MERS patient in the 14 days before symptom onset. For these analyses, we treated all other cases as community-acquired recognizing that this category will include cases resulting from unidentified human-to-human transmission (Appendix Figure 1). Case classification by countries and investigations into the source of infection substantially improved through more systematic data collection since 2015 (*1*), and it is believed that human-to-human cases were more accurately

classified in recent years following the large hospital outbreaks of MERS in Jeddah and Riyadh in 2014.

The large multi-hospital outbreak in Jeddah in 2014 resulted in a total of 255 cases, however, a genomic analysis suggested that this outbreak included multiple introductions from contacts with dromedary camels (*7*). The largest outbreak resulting from one spillover event occurred in ROK in 2015 resulting in 186 cases and 38 deaths, following the return of one individual from the Middle East (*8*).

These analyses were conducted with anonymized case-based data reported to WHO under International Health Regulations and therefore neither informed consent nor approval from an institutional review board was required from WHO.

Simulations

The distribution of human-to-human-transmission cluster sizes observed in 2014–2015 was used to simulate the expected total number of cases in 2016, 2017 and 2018 (through September only), conditional on the incidence of community-acquired cases. The observed numbers were then compared with the distribution of the simulated values to obtain two-sided p-values.

Results

While 739 and 768 cases were reported in 2014 and 2015, respectively, only 244, 244 and 113 cases were reported in 2016, 2017 and 2018* (Appendix Figure 2).

The incidence of community-acquired cases in 2016, 2017 and through September 2018 was 177, 151 and 86, respectively (Appendix Table). These were each highly significantly ($p < 0.001$) lower than expected based on the incidence in 2014–2015 (334, 334 and 251, respectively).

Discussion

Our analysis has several limitations. First, for a virus like MERS-CoV that can cause super-spreading events, it may be challenging to test the hypothesis that human-to-human

transmissibility changed over time because superspreading events can cause high variability in cluster sizes, even in the absence of change. We may therefore have limited power to demonstrate here a significant reduction in human-to-human transmission; analysis of more detailed data capturing the complex transmission dynamics observed in human clusters could prove useful (*9*). Second, classification of cases as human-to-human transmitted or not (community-acquired) depends on the quality of data collection and completeness of the investigations around each case and the time of reporting to WHO. While WHO has regular dialogue with Ministries of Health regarding each case and cluster identified, the follow-up of investigations does not always result in identifying the source of each patient's infection. It is believed that the source of infection was more systematically investigated and reported to WHO since 2015 and that secondary cases resulting from human-to-human transmission were more accurately classified meaning that the distribution of observed cluster sizes in 2014–2015 will be biased toward smaller cluster sizes. This would have the effect of underestimating the number of cases (and deaths) averted due to reduced human-to-human transmission. Third, due to limitations on detailed information about the timing of specific interventions initiated by affected countries and hospitals, we are unable to determine which control measures have made this impact.

Although the trend needs to be confirmed by more detailed analyses, the apparent reduction in human-to-human transmission in healthcare facilities, particularly in the KSA since 2016, and the lack of onward human-to-human transmission in recently exported cases to the United Kingdom (*10*) and the ROK in 2018 (*11*), is likely to be at least partly explained by early suspicion and isolation of cases, immediate case management, improvements in standard infection prevention and initiating control measures and comprehensive identification, follow-up and laboratory testing of all high-risk contacts.

When we look at reported data from 2014/2015, the apparent clusters thus appear smaller on average than was actually true. This also happens 2016–2018*, but to a lesser extent if human-to-human transmission were identified more consistently later. Thus, any cluster size reduction we observe between 2014/2015 and subsequent years is likely an underestimate of the true reduction in human-to-human transmission.

References

1. FAO-OIE-WHO MERS Technical Working Group. MERS: Progress on the global response, remaining challenges and the way forward. Antiviral Res. 2018;159:35–44. PubMed https://doi.org/10.1016/j.antiviral.2018.09.002

2. OIE. Middle East Respiratory Syndrome Coronavirus (MERS-CoV) Case definition for reporting to OIE (Update May 2017) [cited 2019 Jun 4]. http://www.oie.int/en/scientific-expertise/specific-information-and-recommendations/mers-cov/

3. World Health Organization. WHO Updated Guidance: Investigation of cases of human infection with MERS-CoV, Updated June 2018 [cited 2019 Jun 4]. http://www.who.int/csr/disease/coronavirus_infections/mers-investigation-cases/en/

4. World Health Organization. WHO Interim Guidiance: Surveillance for human infection with Middle East respiratory syndrome coronavirus (MERS-CoV), Updated June 2018 [cited 2019 Jun 4]. http://www.who.int/csr/disease/coronavirus_infections/surveillance-human-infection-mers/en/

5. Saeed AA, Abedi GR, Alzahrani AG, Salameh I, Abdirizak F, Alhakeem R, et al. Surveillance and Testing for Middle East Respiratory Syndrome Coronavirus, Saudi Arabia, April 2015-February 2016. Emerg Infect Dis. 2017;23:682–5. https://doi.org/10.3201/eid2304.161793

6. Command and Control Center SAB, Kingdom of Saudi Arabia Ministry of Health. Infection Prevention and Control Guidelines for the Middle East respiratory syndrome coronavirus (MERS-CoV) infection. 4th Edition, 2017 January [cited 2018 Jan 2]. https://www.moh.gov.sa/endepts/Infection/Documents/Guidelines-for-MERS-CoV.PDF

7. Drosten C, Muth D, Corman VM, Hussain R, Al Masri M, HajOmar W, et al. An observational, laboratory-based study of outbreaks of Middle East respiratory syndrome coronavirus in Jeddah and Riyadh, kingdom of Saudi Arabia, 2014. Clin Infect Dis. 2015;60:369–77. https://doi.org/10.1093/cid/ciu812

8. Ki M. 2015 MERS outbreak in Korea: hospital-to-hospital transmission. Epidemiol Health. 2015;37. https://doi.org/10.4178/epih/e2015033

9. Cauchemez S, Nouvellet P, Cori A, Jombart T, Garske T, Clapham H, et al. Unraveling the drivers of MERS-CoV transmission. Proc Natl Acad Sci U S A. 2016;113:9081–6. https://doi.org/10.1073/pnas.1519235113

10. World Health Organization. Disease Outbreak News. Middle East respiratory syndrome coronavirus (MERS-CoV) infection–United Kingdom of Great Britain and Northern Ireland 31 August 2018 [cited 2019 Jun 4]. http://www.who.int/csr/don/31-august-2018-mers-united-kingdom/en/

11. World Health Organization. Disease Outbreak News. Middle East respiratory syndrome coronavirus (MERS-CoV) infection – Republic of Korea 12 September 2018 [cited 2019 Jun 4]. http://www.who.int/csr/don/12-september-2018-mers-republic-of-korea/en

Appendix Table. Reported and expected cases of MERS-CoV infection by year

Year	Reported community-acquired cases*	Expected total cases based on the distribution of case clusters in 2014–2015† (95% range‡)	Expected total cases based on 2014–2015 levels of camel-to-human and human-to-human transmission (95% range‡)	Number of clusters reported by year§	Cluster size by year (range)
2012	6	–		2	2–3
2013	79	–		19	2–25
2014	397	–		10	2–255
2015	271	–		8	2–186
2016	177	398 (189–739)	751 (433–1211)	6	2–33
2017	151	340 (157–663)	751 (433–1211)	19	2–34
2018¶	86	193 (86–451)	564 (304–968)	10	2–12
2016–2018¶	414	931 (566–1420)	2066 (1496–2766)		

*Laboratory-confirmed cases reported to WHO as primary, index or sporadic cases (cases with contact with dromedary camels and without contact with known/probable human MERS cases) and cases under investigation without known contact with another human cases.
†Conditional on reported community-acquired cases
‡The intervals reported in parentheses are the 2.5th and 97.5th centiles of the simulations.
§Data on clusters as reported by WHO Member States and classified into clusters by WHO, and from publications (*3*,*4*,*6*,*7*).
¶Through September 2018.

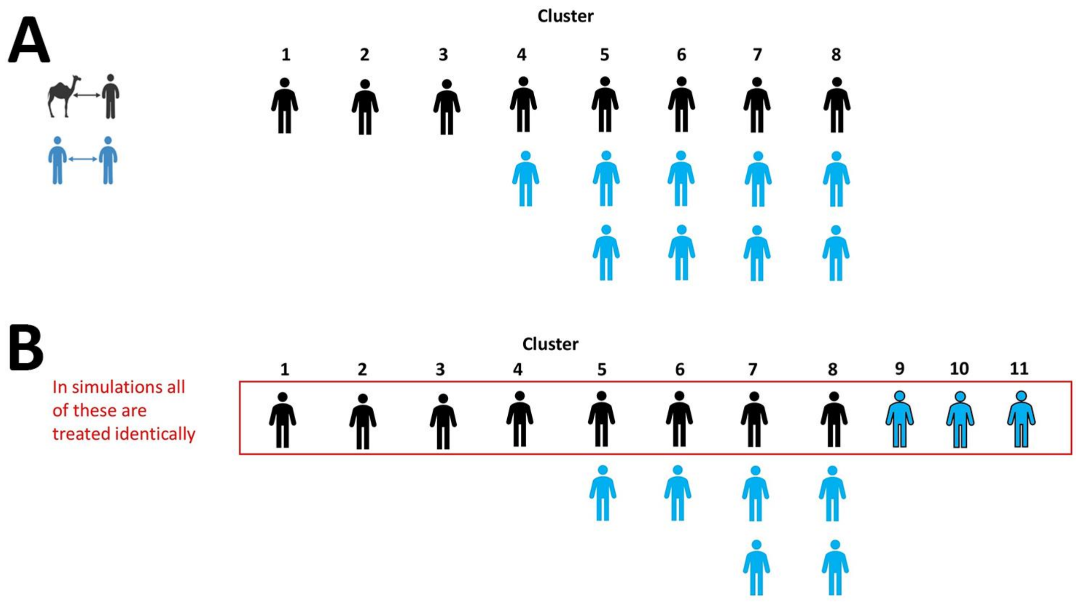

Appendix Figure 1. Weekly incidence of laboratory confirmed MERS-CoV Infection reported to WHO.

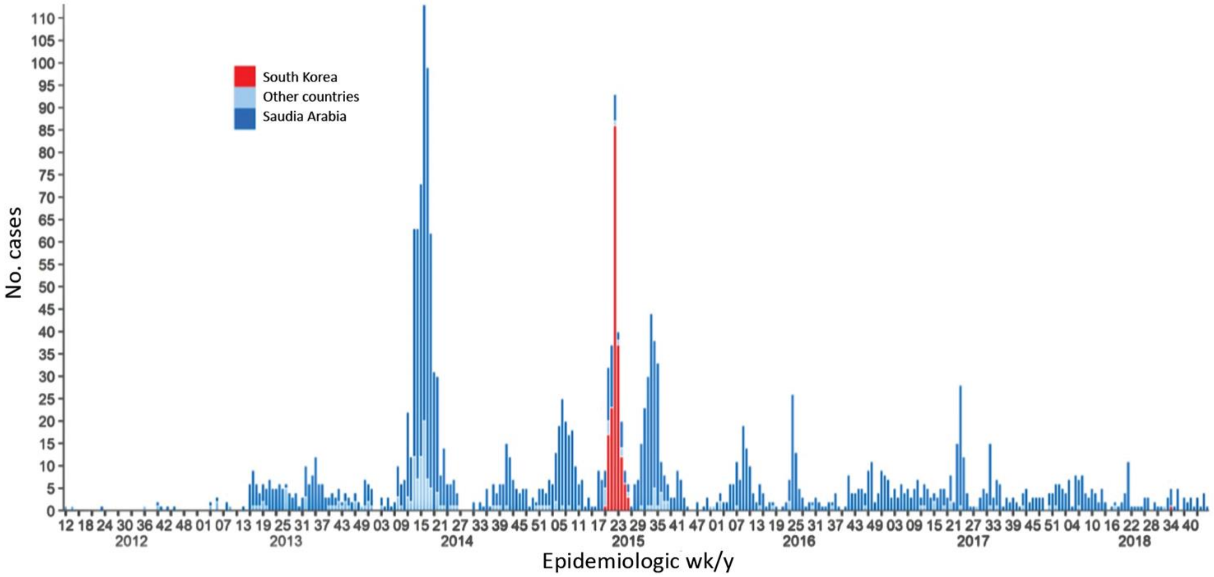

Appendix Figure 2. Clusters of camel-to-human and human-to-human transmission. Panel A Scenario 1: There are clusters – some of size 1 – that all start with a camel-to-human transmission (black). Human-to-human transmission is shown in blue. This shows a situation if all transmission is identified fully. In this scenario, 17 individual cases are reported in 8 clusters and the average cluster size is 17/8, 37.5% (3/8) are single-case clusters and 62.5% (5/8) are clusters of size ≥2 and the average multi-case cluster size is 2.8. In this scenario, reduced human-to-human transmission would only reduce average cluster size. Panel B Scenario 2: There are some clusters where human-to-human transmission is not identified and illustrates that only 5 clusters (numbers 1, 2, 3, 7 and 8) are fully identified. In this scenario, 17 individuals are involved in 11 apparent clusters with an average apparent cluster size of 17/11. Seven of the 11 (63%) are single-case clusters and 4/11 (37%) are clusters of size ≥2 and the average multi-case cluster size is 2.5. In this scenario, reduced human-to-human transmission would appear to reduce both the number of clusters and average cluster size.
